# Continuous Pharmaceutical Manufacturing

**DOI:** 10.3390/pharmaceutics12100910

**Published:** 2020-09-23

**Authors:** Ossi Korhonen

**Affiliations:** PROMIS-Labs, School of Pharmacy, University of Eastern Finland, FI-70211 Kuopio, Finland; ossi.korhonen@uef.fi

Pharmaceutical manufacturing is facing the greatest ideological revolution since industrial drug manufacturing. Drugs have been traditionally manufactured in batch mode, but now, continuous manufacturing has started to gain serious interest in the pharmaceutical industry. The pharma industry is heavily regulated by authorizing bodies (FDA, EMA, etc.), but already, for over a decade, regulatory bodies have enabled, and even encouraged, the change from batch to continuous manufacturing by publishing several guidelines on how to implement continuous manufacturing. The advantages of continuous manufacturing are indisputable. The quality by design framework provides the cornerstone for better process understanding and, ultimately, higher product quality for patients. New modes of the manufacturing of drugs require a substantial amount of research from the pharmaceutical industry, academia, and various research institutions. This Special Issue, “Continuous Pharmaceutical Manufacturing”, obtained six papers covering the wide spectrum of continuous manufacturing. In many papers, modern process modelling or simulation methods were utilized, which facilitate process understanding, development, and, ultimately, advanced process control and automation.

Rehl et al. [1] illustrated how to utilize the modern dynamic process modelling method to create a soft sensor for the moisture content and the end-point prediction of a semi-continuous wet granulation unit operation. In wet granulation, the moisture content in the drying phase is the key critical quality attribute in the decision making of the end-point of the drying phase and the complete wet granulation process. Typically, this is done indirectly following the outlet temperature rise or using constant drying time. However, these indirect measures have shown to be prone to batch-to-batch variability. In this study, model development, parameter identification, and process validation were done with the ConSigma™-25 segmented fluid bed dryer. The study concluded that granule moisture was successfully predicted using just plant input parameters for the soft sensor.

Ervasti et al. [2] studied low dose formulations which tend to segregate in batch-based direct compression processes with the continuous direct compression process (CDC) (powder feeders > continuous blender > tablet press). The universal formulation platform and two low dose (3%) APIs (spironolactone and paracetamol) were studied with the design of experiment (total production rate and blender rpm as process variables). The study concluded that the tablets (with both APIs) fulfilled end product quality requirements without any indication of segregation, even with extreme process settings. Thus, the change in the manufacturing method, from a batch method to its continuous counterpart, could bring benefits when handling this type of formulation.

Van Hauwermeiren et al. [3] created a data-driven modelling framework that links the machine settings of a twin-screw wet granulation unit operation and the output distribution of granules. The knowledge of granule size distribution is relevant information for down-stream unit operation settings. The authors used a two-step procedure: first, the measured distributions are transformed into a high-dimensional feature space, where the relation between the machine settings and the distributions can be learnt. Second, the inverse transformation is performed, allowing an interpretation of the results in the original measurement space. In this approach, they used the kernel mean embedding technique, which is a fast technique and might even allow in-line and real-time prediction of granule size distribution.

Diab and Gerogiorgis [4] went through the recent advances of the continuous flow chemistry of APIs in a review-like paper. They focused especially on how the plantwide design space approach could be used in the continuous synthesis and separation of a wide range of APIs. They included detailed Capital (CapEx) and Operating (OpEx) Expenditure cost components in the models and found them helpful in making a decision on whether to switch from continuous operation to traditional/current manufacturing methods for the API.

Hattori et al. [5] studied a partial least squares regression-based (PLSR) feed-forward control strategy for the tableting process. They used NIR and the physical parameters of granules as input variables to control the lower punch fill depth and the minimum distance between the upper and lower punches on compression, which were specifically related to the tablet’s weight and thickness, respectively. Using high-shear wet granulation, different kinds of granules (particle size distribution, flowability, and loose and tapped density) were prepared to develop feed-forward controls. They successfully demonstrated that robust feed-forward control was feasible using PLSR with the combination of NIR spectra and the physical attributes of the granules to control tablet press settings.

Pauli et al. [6] focused on one of the key aspects of continuous manufacturing: how to manage the start-up phase of unit operation on continuous manufacturing by minimizing the out-of-specification (OOS) material during the start-up phase. This part of continuous manufacturing is challenging due to its transient nature. They demonstrated suitable process control strategies during the start-up of a continuous granulation and drying operation. They focused especially on the start-up of the drying operation and demonstrated that a non-steady process can satisfy the state of the control requirement right from the start without OOS.
